# The Effect of an Exercise Paddock on Dairy Cow Behavior, Health, and Nutrient Digestion during the Transition from Pregnancy to Lactation

**DOI:** 10.3390/ani14162353

**Published:** 2024-08-14

**Authors:** Amin Cai, Shiwei Wang, Pengtao Li, Kris Descovich, Tong Fu, Hongxia Lian, Tengyun Gao, Clive J. C. Phillips

**Affiliations:** 1 College of Animal Science and Technology, Henan Agricultural University, Zhengzhou 450046, China; amincai1105@gmail.com (A.C.); wangshiwei202402@163.com (S.W.); pengtao_li@126.com (P.L.); futong2004@126.com (T.F.); lhx263@sina.com (H.L.); 2 Center for Animal Welfare and Ethics, School of Veterinary Science, Gatton 4343, The University of Queensland, St. Lucia, QLD 4072, Australia; k.descovich1@uq.edu.au; 3 Institute of Veterinary Medicine and Animal Sciences, Estonian University of Life Sciences, Kreutzwaldi 1, 51014 Tartu, Estonia; 4 Curtin University Sustainability Policy (CUSP) Institute, Curtin University, Perth, WA 6845, Australia

**Keywords:** dairy farming, digestion, dry period, exercise paddock, grooming, lying behavior

## Abstract

**Simple Summary:**

In China, dairy cow production has intensified in response to strong demand for milk products. Modern farms house their cows continuously without providing access to pasture, even though there are likely to be health and welfare benefits from the additional exercise. It is most feasible to provide access to an exercise paddock during the time between lactation periods when cows do not need to be brought to a milking parlor each day. We investigated the effects of providing an exercise paddock on the behavior and health of cows during this ‘dry period’. All cows had access to conserved feed indoors, but half were also given access to the exercise paddock. Cows in the group with paddock access spent less time lying down and more time drinking and allogrooming, compared with those in the shed-only group. Lying bouts were shorter in the exercise paddock group. Non-esterified fatty acids, an indicator of a negative nutritional state, increased in cows with the exercise paddock, which may reflect the demands of the increased activity. Fiber and protein digestibility increased with the exercise yard. Overall, our results suggest that providing an exercise paddock was beneficial by increasing physical activity and allogrooming activity during the dry period, with less time spent lying, and increased feed digestibility.

**Abstract:**

Providing an exercise paddock may improve the behavior and health of cows in their dry period. We compared a control group of cows in a shed with no exercise paddock and an experimental group in the same shed but with access to an exercise paddock. Both groups had ad libitum total mixed ration (TMR) indoors combined with access to a paddock (Group EX). The other group was just offered TMR indoors (Group IN). Total lying time was longer for cows without the exercise paddock (859 min/d) than for those with the paddock (733 min/d) (*p* = 0.012). Lying bouts were shorter, there were more allogrooming bouts, and drinking time was longer if an exercise paddock was provided. Cows with the paddock spent on average 76 min/d in paddock activity. Non-esterified fatty acids in the blood were increased by providing the exercise paddock. No significant differences in postpartum milk yield and calf weight of dry cows with or without access to exercise paddock were observed. However, crude protein and neutral detergent fiber digestibility were increased by providing the exercise paddock. The results suggest that providing an exercise paddock for cows in their dry period increased activity, including allogrooming, reduced lying, and improved digestibility of some major nutrients in the feed.

## 1. Introduction

Farm intensification is occurring across most areas of livestock production due to an increasing global demand for animal products, coupled with increasing competition for land from other economic activities, such as crop production, urban development, forestry, biofuel production, recreational land use, mining and extraction, and renewable energy projects [1]. In the dairy industry, intensification has led to an increase in indoor confinement, which promotes a rapid increase in milk production per cow through the provision of high-energy feed and controlled environments [2]. This has required the growing of more high-energy feed to meet the nutritional demands of these high-producing cows [3]. However, indoor housing has had several negative impacts. It can increase the risk of a range of cow health problems [2], decrease behavioral expression [4], and reduce the duration of time that cows remain productive [5]. A number of animal welfare concerns regarding the restrictive nature of intensive dairy cow housing are attracting increased public attention [6].

Dairy systems that incorporate pasture access are likely to have welfare benefits as pasture use is part of a cow’s natural behavioral biology, allowing the expression of normal grazing behavior, which is restricted or eliminated indoors [7]. Pasture and outdoor access also positively influence the expression of important natural behaviors such as social interaction and environmental exploration [8] and reduce the risk of leg health concerns, such as lameness and hock injuries [9,10]. However, it may be challenging for some dairy farms to offer pasture and outdoor access for many reasons. These may arise due to their geographic location or a lack of land availability. Due to variable pasture quality, the ability to manage and provide a consistent feed ration to high-yielding cows, accommodate increases in herd size, and adjust to climatic factors including adverse and unpredictable weather events, may be compromised [11]. In a 2017 study based in the USA, only 34% of dairy operations provided access to pasture or an open area for lactating cows, while 61% of operations provided it for dry cows [12]. In China, dairy farming is primarily conducted through three main modes: intensive-scale farming, pasture grazing, and a mixed model of family farming incorporating animal husbandry [13]. However, since 2008, there has been a trend toward intensification in the dairy cattle farming industry, and currently, the entire industry is shifting its focus toward increasing scale [14]. The percentage of farms with dairy cattle numbers that exceed 1000 heads increased from 23.6% in 2014 to 43% in 2019 [13]. At the same time, the provision of exercise yards has decreased, with many new dairy farms not providing any exercise yards. The proportion of dairy farms with exercise yards has decreased substantially, from 96.4% in 2016 to 59.2% in 2020 [15]. 

The dry period is a crucial phase within the dairy cattle production cycle, during which cows are very vulnerable to health issues. During this phase, cows cease milk production, allowing their mammary glands to rest and recover while establishing reserves of fat and protein for the subsequent lactation cycle [16]. At the same time, cows experience reduced metabolic demands and decreased nutritional turnover, alongside improvements in their digestive and immune systems [17]. However, the metabolic transition from the lactating period to the dry period predisposes dry cows to certain health issues [18]. Prior research has identified several welfare concerns for dry cows: increased experience of negative/unpleasant emotions such as frustration induced by sudden cessation of milking, hunger resulting from dietary changes, and discomfort from engorgement of the mammary gland during the drying-off process, inadequate comfortable resting areas, and insufficient cleanliness of bedding during parturition [19,20]. Cows in this phase require specific attention to husbandry and the environment, for example, the provision of adequate and comfortable resting areas, and clean, hygienic bedding during parturition [19,20]. 

Dairy cows are known to benefit from access to the outdoors. Outdoor access, sunlight exposure, and more than two hours of exercise (walking) a day are conditions known to benefit dairy cow health, resulting in improved muscle tone [21], and reduced risk of abnormal positioning of the fetus, placenta retention, calving difficulties, and hoof disease [11,22]. Exposure to the sun promotes the synthesis of vitamin D, which helps to prevent postpartum paralysis and placenta retention [23,24]. Where there are concerns that outdoor access will jeopardize the nutrient intake of dairy cows, this can be managed by ensuring that exercise paddocks contain little herbage and by providing a full nutrient supply indoors [25]. This combined system is common in countries where the minimum dairy cow legislated requirements stipulate that access to an exercise area must be provided in order to ensure adequate animal welfare [26]. 

The aim of this study was to investigate whether the provision of an exercise paddock affected the welfare and production of cows during their dry period, as evidenced by cow behavior, serum metabolites, apparent digestibility, and productivity.

## 2. Materials and Methods

### 2.1. Animals, Housing, and Management

This study was conducted at the Henan Ruiya Animal Husbandry Co., Ltd., Dairy Farm, Xincai County, Zhumadian City, Henan Province, China, from April to June 2020. The study period commenced approximately 55 days before parturition and continued until the cows gave birth. All procedures involving animals were approved by the Animal Ethics Committee of Henan Agricultural University under the Guidelines for Ethical Review of Laboratory Animal Welfare (GB/T35892). 

**Figure 1:**
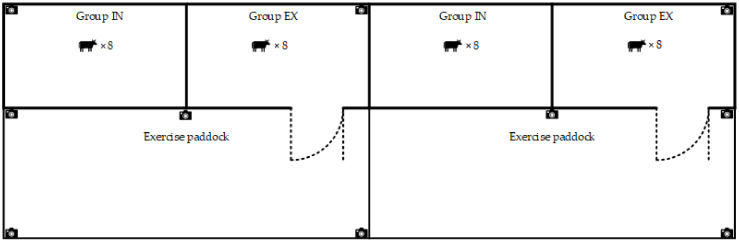
Overhead elevation of the cow shed. Group IN = indoor group with no exercise paddock, and Group EX = Group with an exercise paddock. 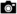
= camera.

A 210 × 18 m experimental shed was used for the study, which was divided into two halves for replicates of the treatments. The shed was a portal-framed building, with a double-sloping roof design of colored steel tiles, sides consisting of a steel frame and sheeting, and a concrete floor. The shed was equipped with bedding, which consisted of a mixture of dried manure, rice husks, and peanut shells. The dried manure was produced on the farm, using an automatic scraper to push the manure from the barn floor to an underground storage pit at one end of the barn. Under the action of a pressure pump, the manure was transported to the manure treatment area where it immediately underwent mechanical solid–liquid separation. The separated solid manure was not fermented and was directly air-dried until its moisture content reached 3.62 ± 0.31%. The ends of the shed were in the east/west direction, and the shed was naturally lit for approximately 11.8~13.5 h each day. An outdoor exercise paddock (50 × 20 m) was located adjacent to the shed and connected via the shed’s open sides. The paddock had steel pipe fencing and a soil base. Both the shed and exercise paddock were divided into four adjacent pens by a steel pipe fence (Figure 1). The exercise paddock was naturally lit for approximately 11.8–13.5 h each day, and it had no water facilities, feeding facilities, or a roof for shade.

Ambient temperature and humidity were monitored every two hours between 06:00 h and 20:00 h using temperature and humidity loggers (Xiaomi MIJIA, LYWSDCGQ/01ZM, Beijing, China) positioned out of direct sunlight and at the approximate height of a cow’s body. Three temperature/humidity loggers were positioned in the shed and three were in the exercise paddock. The mean daily temperature in the shed was 25.2 ± 6.3 °C, ranging from 17.2 to 31.9 °C, with humidity averaging 66.8 ± 19.7% and ranging from 39% to 82.7%. In the exercise paddock, the mean temperature was 27.9 ± 7.2 °C, ranging from 17.0 to 39.0 °C, and the humidity ranged from 17.7% to 64.3% with a mean of 38.0 ± 14.8%.

Thirty-two non-lactating and pregnant (mean 55 ± 7d before parturition) Holstein dairy cows between 3 and 4 years old (second parity) were divided into two treatment groups of 16 cows per group, based on calving date and milk yield before dry-off, with 16 cows in each group (IN, no exercise paddock, and EX, with an exercise paddock). Each group consisted of two replicates, with 8 cows in each replicate. The shed and exercise paddock were divided into four adjacent pens by a steel pipe fence, as illustrated in Figure 1.

Cows were fed a total mixed ration (TMR) at 06:00 h and 18:00 h. The TMR contained 38.4% corn silage, 15.4% wheat straw, 13.4% oat hay, 13.6% concentrate mixture (with crude protein ≥20%, Ca 0.5–1.0%, P 0.5–0.7%, and Lys 0.8%), and 19.2% water on a fresh weight basis (added to achieve the desired moisture content). On a dry matter basis, the TMR contained 23.2% corn silage, 27.5% wheat straw, 24.2% oat hay, and 25.1% concentrate mixture. Ad libitum access to water was provided in a trough in each section of the shed. The majority of births were assisted by a veterinarian. Dystocia in the cows was not recorded. Calf birth weight was recorded using an electronic balance (TCS-150, Huifeng, China). After parturition, cows were taken to the milking parlor at three fixed times daily for milking: between 05:00 and 06:00 h, between 15:00 and 16:00 h, and between 23:00 and 24:00 h. Milk yield for the first month after calving was recorded from each cow.

### 2.2. Video Observations

The behavior of the cows was monitored using ten high-definition digital video cameras (MC-8624-H2-400 W, MA CA (China) Co., Ltd., Shenzhen, China) installed on columns in the shed and exercise paddock, as illustrated in Figure 1. A digital video recorder (MC-8809-K1, MA CA (China) Co., Ltd., Shenzhen, China) was attached to these cameras for continuous, 24 h recording of the behavior. Cameras contained built-in infrared lights for night-time recordings. The first seven days of the experiment were considered to be an adjustment period for the cows, during which time the behavior was not recorded. From day 7 until day 60, the behavior was coded from the video recordings by a single trained observer using focal animal sampling with continuous recording. Individual cows were distinguished by their unique coat color patches, and each cow was marked with animal marking crayons to indicate their identification numbers. The following were recorded: the total duration of lying down, the frequency of bouts of lying down, the mean duration of lying bouts, eating and drinking, the duration of time spent in the exercise paddock, and auto- and allogrooming (Table 1).

### 2.3. Serum Indicators

Blood samples were drawn from the coccygeal vein of each cow within 4 h of calving. Blood was collected into 5 mL tubes without anticoagulants and centrifuged at 4000× *g* for 10 min, and the resulting serum collected in a sterile 1.5 mL centrifuge tube and stored at −20 °C until analysis. Serum samples were analyzed for serum non-esterified fatty acid (NEFA), serum total calcium (Ca), and B-hydroxybutyrate (BHB), using a fully automated bio-analysis machine (AU5800, Beckman Coulter, Brea, CA, USA). 

### 2.4. Apparent Total-Tract Digestibility

Fecal grab samples (400 g per sampling) were collected from the rectum of each cow at 0800, 1200, 1600, and 2000 h (d 1), and at 0200, 0600, 1000, 1400 h, 1800 h, and 2200 h (d 2), and 0000, 0400, and 0800 h (d 3), over 3 consecutive days during week 8 of the treatment period. A total of 96 fecal samples were collected from each cow, at each time interval, every day and pooled in equal proportions, resulting in a final mixture of 600 g per portion. During the same sampling period, feed samples were collected daily using a five-point sampling method at the feed channel after feeding the cows at 06:00 h and 18:00 h, and aggregated feed samples taken on one day for each group over 3 d, making a total of 12 feed samples, were collected. All feed samples were analyzed for apparent total-tract digestibility of dry matter (DM), crude protein (CP), neutral detergent fiber (NDF), acid detergent fiber (ADF), and ether extract (EE), with reference to the acid-insoluble ash (AIA) in accordance with GB/T23742-2009 (China) [30]. In vivo total-tract nutrient digestibilities were calculated from AIA and nutrient concentrations in the orts-adjusted diet and feces:

Apparent digestibility of a nutrient in the feed (%) = 100 × [1 − (AIA content in the feed/AIA content in the feces × nutrient content in the feces/nutrient content in the feed)].

### 2.5. Statistical Analysis

Individual cows were considered the observational unit. After performing the Anderson–Darling test for normality and Bartlett’s test for homogeneity of variances, we chose a mixed-effects model for analysis, with treatment as the primary fixed effect, replicate as the secondary fixed effect, and individual animals as random effects. The analysis was conducted in Minitab, and the average reliability was 0.95. A statistical significance was considered to have occurred at *p* < 0.05.

## 3. Results

### 3.1. Behavior

**Figure 2:**
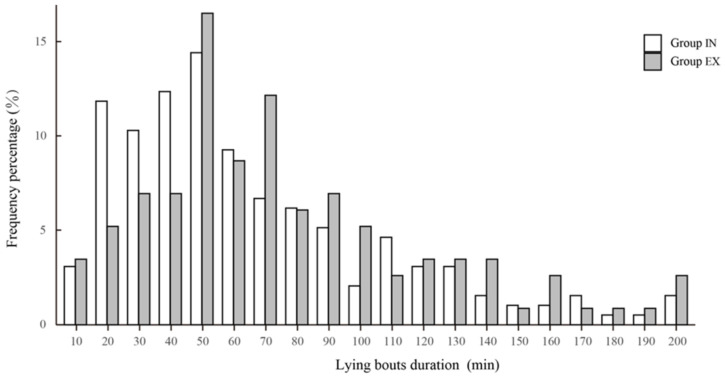
Frequency percentage distribution of cow lying duration for cows in Treatments IN and EX. Group IN = indoor group with no exercise paddock, and Group EX = Group with an exercise paddock.

Cows with outdoor access spent means for the two replicates of 75.92 ± 27.85 and 76.44 ± 23.84 min/d in their exercise areas. Cows in treatment EX spent less time lying than cows in treatment IN, and lying bouts were shorter (Table 2). There were no differences in the number of lying bouts. The highest proportion of lying durations in both groups of cows clustered around approximately 50 min, cows in Group EX primarily exhibited lying durations between 20 and 50 min, whereas those in Group IN predominantly showed lying durations between 50 and 70 min (Figure 2).

Cows in treatment EX tended to stand for longer than those in treatment IN (*p* = 0.085), but there was no difference in eating times. Drinking time was significantly higher in the treatment EX group compared with Group IN. Cows in treatment EX spent significantly more time allogrooming than those in treatment IN (*p* < 0.05), but there was no significant difference in the bouts of autogrooming between the two treatments.

### 3.2. Serum Metabolite

Serum metabolite concentrations were all within the normal range. The serum NEFA concentration was significantly higher in Group EX compared with Group IN, but there were no significant differences between treatments for the other serum metabolite concentrations (Table 3). There were no significant differences in calf weight or postpartum milk yield when the exercise paddock was provided for dry cows.

### 3.3. The Total-Tract Apparent Digestibilities

The total-tract apparent digestibilities are presented in Table 4. The apparent digestibility of CP and NDF was significantly higher in Group EX compared with Group IN (*p* < 0.05). There were no significant group differences in the apparent digestibility of DM, ADF, and EE (*p* > 0.05).

## 4. Discussion

### 4.1. Behavior

The expression of natural behaviors such as feeding, drinking, lying, and standing are key indicators of cow welfare because the expression of natural behaviors is considered to be a preference test for whether animals are in rich natural environments, which meets the basic needs for animal survival [23]. In this study, the total lying time of cows in the exercise group (733 min/d) was lower than that in Group IN (859 min/d); however both groups performed lying behavior in amounts sufficient to support good welfare (12–14 h/d) [31]. Lying time in the EX cows was largely replaced by activity in the exercise paddock, suggesting that the cows were more motivated to exercise and explore due to their increased space allowance. It is already known that increasing cow movement and grazing during the dry period can reduce the subsequent discomfort of dairy cows during parturition [32], and further research should investigate the effects of the exercise paddock on ease of parturition. 

The exercise paddock increased activity but did not decrease lying behavior to an unhealthy level, which suggests improved welfare in the experimental cows. However, differences were noted in the mean bout length of lying behavior. Charlton et al. and Westin et al. [33,34] reported averages of 9 to 11 lying bouts/d, with mean bout duration varying from 60 to 99 min. In this study, the number of lying bouts in Group EX (11.9 bouts/d) was not significantly different from Group IN (10.5 bouts/d); however, mean lying bouts durations were shorter in Group EX (66 min) than those in Group IN (85 min). Although both groups exhibited the highest proportion of lying durations around 50 min, cows in Group EX predominantly had lying durations between 20 and 50 min, whereas cows in Group IN typically ranged between 50 and 70 min. This is attributed to increased motivation for activity resulting from the provision of outdoor exercise opportunities [35]. Cows at pasture typically have shorter lying times compared with those housed indoors. However, despite the reduced total lying time, cows often prefer lying on pasture because of the greater comfort; they occupy a greater variety of lying postures, which enhances their welfare [36]. Housing systems that offer more movement opportunities, such as when pasture or an exercise yard are included, enable cows to be more active and avoid exercise deficiencies that can occur in confined spaces [37]. This increased activity can lead to more frequent standing, walking, and lying down, which, in turn, increases the frequency of lying bouts but shortens the duration of each bout, attributable to the cows’ ability to move freely in a larger space, thereby improving their overall welfare [37].

Allogrooming improves relationships between animals [28]. Positive social behaviors such as sniffing, body rubbing, and autogrooming behaviors between dairy cows, are associated with reduced amounts of inter-cow aggression [29]. In contrast, housed feeding dairy cows often show more autogrooming, although no difference was found in the current study for this behavior. Autogrooming can develop into a stereotypical behavior and may be associated with negative emotional states of boredom, frustration, or increased stress in dairy cows [38]. While autogrooming was not affected by treatment in this study, the provision of an exercise paddock increased allogrooming behavior. In combination with the increase in the exercise behavior, this suggests that the paddock had an important positive impact on the behavior of the cows. Smid et al. [36] reported that outdoor areas, particularly pastures, help increase positive social behaviors among dairy cows, such as allogrooming.

### 4.2. Serum Metabolites

Most cows experience a period of negative energy balance immediately after calving due to reduced pre-calving feed intake and increased energy requirements for milk production [39,40]. A successful transition from pregnancy to lactation requires a series of complex and coordinated changes in metabolism and nutrient partitioning [41]. Failure of these transitions can lead to metabolic disorders, such as ketosis and fatty liver [42]. These diseases have a significant negative effect on the health and welfare of early-lactation dairy cows [43]. Serum β-hydroxybutyrate (BHB) and non-esterified fatty acids (NEFA) are biomarkers that are commonly used to evaluate the energy balance of dairy cows in the transition period [44,45,46]. Serum NEFA concentration is a measure of the degree of negative energy balance [47]. BHB, on the other hand, is commonly used as a biomarker of energy balance [48]. In the current study, there were no significant effects of providing an exercise paddock on postpartum serum BHB concentrations, and serum NEFA levels were significantly increased in cows in treatment EX, but the mean BHB and NEFA serum levels were within the normal reference range [49,50]. The increased serum NEFA levels in the exercise paddock group could be related to increased energy expenditure in this group. Exercise increases the energy demand for muscle activity, prompting the breakdown of adipose tissue to provide additional energy sources. This finding is consistent with Reynolds et al.’s research, which found that exercise can increase the rate of fatty acid mobilization in dairy cows, thereby raising serum NEFA concentrations [51]. Previous research indicates that changes in feeding behaviors and walking activity during the transition period are associated with subclinical ketosis (SCK) during the week after calving [52,53]. Itle et al. [54] recently found that cows with SCK after calving spent less time lying in the week before calving. This evidence suggests that feeding behaviors, dry matter intake, and lying behavior play an important role in transition cow health. Access to an exercise paddock for dairy cows during the dry period, which decreased the total lying time and increased paddock activity times, may be beneficial to cows’ metabolism. 

Venjakob et al. [55] defined subclinical hypocalcemia as a serum calcium concentration of less than 2.0 mmol/L (8.0 mg/dL) within the first two days after calving. Increased paddock activity times may have the potential to promote bone calcium mobilization in dairy cows, but the specific effects need to be determined in further studies. The maternal environment plays a significant role in fetal growth and development during gestation. Black [56] reported that physical activity tended to affect birth weight, with calves weighing 39.2 ± 5.4, 42.1 ± 6.8, and 44.3 ± 6.3 kg when born to confined, exercise, and pasture cows, respectively. In general, milk yield is negatively correlated with lying time [57]. In this study, total lying time was less in Group EX than in Group IN, but milk yield was not significantly different, suggesting that providing exercise paddocks for dry cows does not adversely affect postpartum performance despite increased NEFA at calving.

### 4.3. The Total-Tract Apparent Digestibilities

Several factors affect the digestive efficiency of cows, such as diet composition, associative effects among individual feeds in the diet, and rate of digesta passage through the digestive tract [57]. Dietary composition and nutrient content are critically important. Exercise and physical training are also known to affect gastrointestinal function and digestibility in horses [58,59,60,61]. Bergero et al. [59] observed that DMD digestibility and organic matter digestibility decreased with increasing workload. However, the treatment effects on nutrient digestibility could not entirely be attributed to exercise [60,61]. The level of DM intake might have a confounding effect on the digestibility of dietary components [62]; increasing feed intake reduces diet digestibility when cows are fed the same diet at maintenance versus productive levels of feed intake [63]. In this study, the feeding times and diet composition of cows in the two groups were the same, suggesting similar intakes. Despite this, CP and NDF digestibility were increased in cows with the exercise paddock, which may be due to improved gastrointestinal tract motility. Previously, it has been determined that NDF digestibility is increased in working oxen during 3-month periods of work, compared with the period of approximately 40 d before and after work [64]. CP digestibility was also greater during the working period compared with beforehand. In another study, exercised donkeys tended to have increased feed digestibility compared with resting donkeys [65]. 

## 5. Conclusions

The provision of an exercise paddock increased outdoor activity and allogrooming behavior in dry dairy cows. Lying behavior decreased but remained within recommended limits. Non-esterified fatty acids in the cows’ blood at calving increased with the exercise paddock, which may reflect the nutritional demands of increased activity. Digestibility of feed neutral-detergent fiber and crude protein increased with the exercise paddock, which may be beneficial during lactation. Further research is needed to confirm the long-term effects of providing exercise paddocks for dry cows on their cow health, welfare, and production, as well as the occurrence of postpartum diseases in cows, and the growth and health of calves.

## Figures and Tables

**Table 1:** Ethogram of cows recorded during the experiment.

Behavior	Description [27,28,29]
Lying down movement	Begins once the cow bends its front carpal joint and lowers the body and ends when the hindquarter of the cow is completely down and the cow pulls the front leg out from underneath the body.
Lying time	Starts once the ‘Lying down movement’ is complete and ends once the ‘Getting up movement’ commences.
Getting up movement	Begins when the cow lifts the hindquarter from the ground and ends when both front legs touch the ground and the whole-body weight is distributed across all four legs.
Eating	The muzzle or head of the cow is in or over the feed bunk.
Drinking	The muzzle or head of the cow is in or over the water trough.
Paddock activity	Begins when the cow enters the paddock for standing or walking and ends when the cow leaves the paddock.
Allogrooming	Licking movements by one cow carried out on the body of another
Autogrooming	Licking the cow’s own body

**Table 2:** Dairy cow behavior (means ± standard deviation) and the effect of providing an exercise paddock (means).

Item	Group EX	Group IN	SED	*p*-Value
Total lying time, min/d	732.6	858.8	24.43	0.000
Number of lying bouts, bouts/d	11.9	10.6	0.94	0.232
Mean lying bout duration, min	66.2	85.5	8.16	0.025
Standing, min/d	364.4	323.5	22.27	0.085
Eating, min/d	239.5	244.9	17.41	0.766
Drinking, min/d	7.2	4.9	0.52	0.000
Paddock activity, min/d	76.18	-	-	-
Allogrooming, bouts/d	5.0	0.8	0.31	0.000
Autogrooming, bouts/d	4.2	4.45	0.48	0.661

Treatment EX = cows with an exercise paddock; treatment IN = cows only indoors without an exercise paddock. Different superscript letters within a row indicate *p* < 0.05. Group IN= indoor group with no exercise paddock, and Group EX = Group with an exercise paddock.

**Table 3:** Effects of providing exercise paddock on mean values for serum metabolites at calving, calf weight, and milk yield in the month after calving.

Measure	Group EX	Group IN	SED	*p*-Value
BHB, mmol/L	0.8	0.7	0.06	0.271
NEFA, mmol/L	1.2	1.0	0.11	0.031
Ca, mmol/L	2.0	2.0	0.07	0.291
Calf weight, kg	38.1	37.8	1.16	0.411
Postpartum milk yield, kg/d	35.6	34.5	1.28	0.473

BHB = β-hydroxybutyrate; NEFA = non-esterified fatty acids; Ca = calcium. Group IN= indoor group with no exercise paddock, and Group EX = Group with an exercise paddock.

**Table 4:** Differences in apparent digestibility of dairy cows between groups.

Apparent Digestibility	Group EX	Group IN	SED	*p*-Value
DM, %	65.2	63.6	1.82	0.390
CP, %	71.3	66.0	1.94	0.014
NDF, %	53.3	49.8	1.57	0.041
ADF, %	44.4	43.4	1.60	0.519
EE,%	83.4	79.6	2.36	0.131

DM = dry matter; CP = crude protein; NDF = neutral detergent fiber; ADF = acid detergent fiber; EE = ether extract; Group IN= indoor group with no exercise paddock, and Group EX = Group with an exercise paddock.

## Data Availability

The data used in this study are available from the corresponding author on request.
